# Genome-Wide Association Analysis of the Sperm Motility Traits of Jinding Drakes

**DOI:** 10.3390/ani16111694

**Published:** 2026-06-01

**Authors:** Chunhong Zhu, Haotian Gu, Zhicheng Wang, Weitao Song, Zhiyun Tao, Shuangjie Zhang, Li Chen, Huifang Li, Hongxiang Liu

**Affiliations:** 1 Jiangsu Institute of Poultry Science, Yangzhou 225125, China; zhuch_1304428@126.com (C.Z.); guhaotian1998@163.com (H.G.); wzcjips@126.com (Z.W.); weitaosong@126.com (W.S.); zhiyun2@126.com (Z.T.); zhang0101@sina.com (S.Z.); 2 Zhejiang Academy of Agricultural Science, Hangzhou 310021, China; chenli@zaas.ac.cn

**Keywords:** sperm motility parameter, genome-wide association study, single-nucleotide polymorphisms (SNPs), duck, candidate gene

## Abstract

Computer-assisted sperm analysis was conducted to phenotypically and genetically assess the sperm motility traits of Jinding drakes. The phenotypic evaluation revealed moderate variability across motility parameters, consistent with a polygenic inheritance pattern. Correlation analysis further demonstrated strong associations among velocity-related traits and inverse relationships between linearity and lateral head displacement metrics. Genome-wide association studies identified 15 significant single-nucleotide polymorphisms (SNPs) associated with five key sperm motility traits (straight-line velocity, curvilinear velocity, average path velocity, amplitude of lateral head displacement, and mean angular displacement) at a genome-wide threshold of *p* < 1 × 10^−6^. Notably, of these 15 SNPs, nine were concentrated on chromosome 1, indicating the presence of a genomic hotspot for regulation of sperm motility. Pleiotropic effects were evident, as several SNPs were found to influence multiple motility traits. Candidate genes implicated in essential sperm functions included *Myo16* (cytoskeletal dynamics), *Cep76* (flagellar structure), and *Jarid2* (epigenetic regulation during spermatogenesis), as well as genes involved in membrane integrity, mitochondrial function, and immune regulation. These findings provide novel insights into the genetic architecture underlying sperm motility of Jinding drakes and establish a basis for molecular breeding strategies to enhance reproductive efficiency of waterfowl.

## Simple Summary

This study used computer-assisted sperm analysis to examine sperm movement traits in Jinding drakes. The results showed moderate variation in swimming ability among individuals, suggesting that these traits are controlled by many genes rather than a single gene. Certain movement characteristics, such as speed and wiggling patterns, were strongly linked to each other. By scanning the ducks’ genomes, researchers identified 15 specific genetic markers (SNPs) linked to five key sperm motility traits. Most of these markers were found on chromosome 1, pointing to a hotspot region for sperm function. Several markers influenced more than one motility trait, indicating shared genetic control. Candidate genes near these markers are involved in sperm structure, energy production, and development. These findings help explain the genetic basis of sperm motility in ducks and provide useful tools for breeding programs aimed at improving reproductive success in waterfowl.

## 1. Introduction

As a vital component of male fertility across species, sperm motility determines the ability of sperm to traverse the female reproductive tract and reach the egg for successful fertilization. Key motility parameters such as straight-line velocity (VSL), curvilinear velocity (VCL), average path velocity (VAP), and amplitude of lateral head displacement (ALH) have been linked to fertility outcomes in various species, including ducks. Despite their importance, the genetic mechanisms underlying these traits in ducks are not well characterized.

As a distinguished indigenous egg-layer breed in China, Jinding drakes exhibit outstanding reproductive performance, including early sexual maturity, high fertilization rate, and excellent semen quality, making them highly efficient for both natural mating and artificial insemination. In poultry breeding, they serve as an elite paternal line for supporting purebred conservation, cross improvement, and the breeding of high-efficiency green-shell egg duck supporting lines.

Recent advancements in genomic technologies, particularly genome-wide association studies (GWAS), have enabled the identification of candidate genes associated with sperm motility traits of livestock and other species. For example, in cattle, specific single-nucleotide polymorphisms (SNPs) on chromosomes 1, 2, and 6 have been linked to VSL and related sperm motility traits, leading to the identification of key genes associated with sperm development and function [1]. The genes *Ptpn2* and *Cep76* are reportedly critical for sperm function and motility of both farmed and wild pigs [2] as well as rodents [3]. A prior GWAS found that the candidate genes *Psmb5*, *Prmt5*, and *Actb* were associated with semen volume, sperm concentration, and sperm motility of Chinese Holstein cattle [4], while a more recent GWAS revealed novel loci and candidate genes related to the body size, growth rate, and carcass yield of ducks, thereby providing a theoretical basis for genetic improvement [5]. These studies underscore the importance of identifying genetic markers for selective breeding programs to improve the reproductive efficiency of poultry.

Semen quality is a key determinant of reproductive success in duck breeding. Although several studies have investigated the genetic architecture of semen traits in ducks, knowledge regarding genes associated with sperm motility remains limited. Accordingly, this genome-wide association study (GWAS) was performed to identify genetic markers and candidate genes linked to sperm motility traits in ducks. Research on semen quality in Jinding drakes is critical for improving artificial insemination efficiency, maintaining high fertilization rates, and preserving the genetic superiority of this elite breed in modern duck breeding programs.

## 2. Materials and Methods

### 2.1. Animals and Study Approval

Adult Jinding drakes (n = 160) were raised in individual cages under identical environmental conditions with *ad libitum* access to feed and drinking water. The study protocol was approved by the Animal Care and Use Committee of the Jiangsu Institute of Poultry Science (approval no. JIPSAC20241203) and conducted in accordance with established regulations and guidelines.

### 2.2. Semen Collection and Quality Assessment

Semen was collected via stimulated ejaculation into tubes, which were immediately placed in an ice bath at 4 °C. Each duck was subjected to semen collection twice at a 4-day interval, and all valid ejaculates from each individual were used for subsequent detection. Sperm motility parameters were assessed within 2 h using a MAILANG ML-608JZ poultry-specific computer-assisted sperm analysis (CASA) system (MAILANG, Nanning, China), equipped with a 20× objective and a thermostatic stage maintained steadily at 37 ± 0.3 °C. Raw semen was isothermally diluted with pre-warmed (37 °C) duck dedicated semen extender to a final sperm concentration of 30–40 × 10^6^ sperm/mL. A 5 μL aliquot of diluted semen was loaded into a matched MAILANG disposable counting chamber and allowed to stabilize for 30 s before detection. All samples were analyzed in a fixed processing order to guarantee consistent waiting time between semen collection and detection across individuals, with only negligible time differences among samples. For each sample, five random microscopic fields were captured, and no fewer than 800 valid spermatozoa were analyzed. The analysis time for each individual sample was controlled within 10 s. Unified system settings were applied throughout the experiment: sperm head area of 20–70 μm^2^, minimum progressive velocity threshold of 5 μm/s, valid track length ≥ 10 frames, and velocity detection range of 0–250 μm/s. The recorded motility parameters included VSL, VCL, VAP, ALH, and MAD, in addition to wobble (WOB), linearity (LIN), and straightness (STR). All CASA procedures were strictly standardized to eliminate measurement-induced variation, ensuring that phenotypic differences reflected genetic variation for subsequent GWAS analysis.

### 2.3. DNA Extraction and Whole-Genome Sequencing

Blood samples were collected by sterile venipuncture from the wing brachial vein of Jinding ducks. Genomic DNA was extracted from blood samples collected from each duck using the MagAttract HMW DNA Kit (Qiagen GmBH, Hilden, Germany) and assessed with a NanoDrop 2000 spectrophotometer (Thermo Fisher Scientific, Waltham, MA, USA). Qualified samples were subjected to whole-genome sequencing with the T7 platform (depth, 10×). SNP calling was performed using the Genome Analysis Toolkit (https://gatk.broadinstitute.org/) (accessed on 12 August 2025) in reference to the ZJU1.0 duck genome assembly (https://www.ncbi.nlm.nih.gov/datasets/genome/GCF_015476345.1/) (accessed on 13 August 2025).

Quality control of samples and SNPs was performed using the PLINK open-source whole-genome association analysis toolset (v1.9; https://www.cog-genomics.org/plink/) (accessed on 13 August 2025), while excluding sequences that failed to meet one or more of the following conditions: sample call rate < 95%, SNP call rate < 90%, Hardy–Weinberg equilibrium (HWE) test *p*-value ≤ 0.001, and minor allele frequency (MAF) < 0.05.

### 2.4. GWAS

The SNPs were excluded that (i) had a MAF lower than 0.05, (ii) deviated from HWE (*p*-value ≤ 0.001), and (iii) for which there were more than 5% missing genotypes. Principal component analysis (PCA) was conducted based on SNPs to assess population genetic structure. SNP-trait association analysis was performed using a univariate linear mixed model based on all SNPs with GEMMA software (v0.98.4; https://github.com/genetics-statistics/GEMMA/releases) (accessed on 14 August 2025). The statistical model was calculated as Yilm = μi + ki + Gm + eilm, where Yilm is the phenotypic value, μi is the common mean, ki is the kinship matrix, Gm is the effect of the SNP, and eilm is the random residual. The trait values were treated as numerical. The significance of the associations was determined with the Wald test. Based on a previous principal component analysis (PCA) for a population of ducks, the corresponding number of principal components was not included in the model. Significant SNPs were defined as the genome-wide threshold of *p* < 1 × 10^−6^. The false discovery rate (FDR) approach was used for multiple testing correction; all SNPs meeting *p* < 1 × 10^−6^ also satisfied FDR ≤ 0.05 were kept for further analysis. Manhattan plots and quantile-quantile plots for the GWAS results were generated with the R package “CMplot” (version 4.2.3; https://www.r-project.org/) (accessed on 14 August 2025).

### 2.5. Candidate Gene Analysis and Functional Annotation

For identification and functional characterization of candidate genes, the genomic regions’ flanking significant SNPs (±100 kb) were extracted with the Bedtools software suite (v2.30.0; https://bedtools.readthedocs.io/en/latest/) (accessed on 15 August 2025) and annotated using the Ensembl bioinformatics database (https://www.ensembl.org/index.html) (accessed on 15 August 2025) and University of California–Santa Cruz Genome Browser (https://genome.ucsc.edu/) (accessed on 15 August 2025). Kyoto Encyclopedia of Genes and Genomes (KEGG) analyses were performed based on candidate genes for each trait via the Database for Annotation, Visualization, and Integrated Discovery (DAVID, Version 6.8, https://davidbioinformatics.nih.gov/) (accessed on 15 August 2025).

### 2.6. Statistical Analysis

All experimental data were analyzed using IBM SPSS Statistics for Windows (version 25.0; IBM Corporation, Armonk, NY, USA) and Pearson correlation coefficients were calculated.

## 3. Results

### 3.1. Phenotypic Data of Sperm Motility Quality Traits

The descriptive statistics including average values and coefficients of variation in the eight motility parameters of all 160 Jinding drakes evaluated in this study are presented in Table 1. These results provide a comprehensive phenotypic overview of the sperm motility characteristics of Jinding drakes, reflecting both central tendency and individual variability in sperm motion dynamics.

### 3.2. PCA of Sperm Motility Parameters of Jinding Drakes

**Figure 1:**
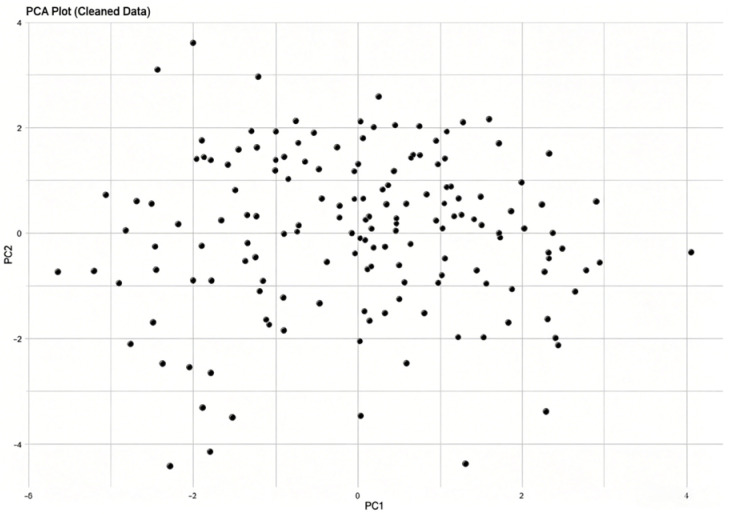
PCA of sperm motility parameters of Jinding drakes.

PCA was performed to assess the variation and relationships among the eight semen quality traits across 160 samples. The two-dimensional scatter plot presented in Figure 1 shows a dispersed distribution of individuals along PC1 and PC2 with no clear clustering pattern, indicating substantial individual variation in sperm motility traits but no evident population substructure.

### 3.3. Correlation Analysis of Sperm Motility Parameters

**Figure 2:**
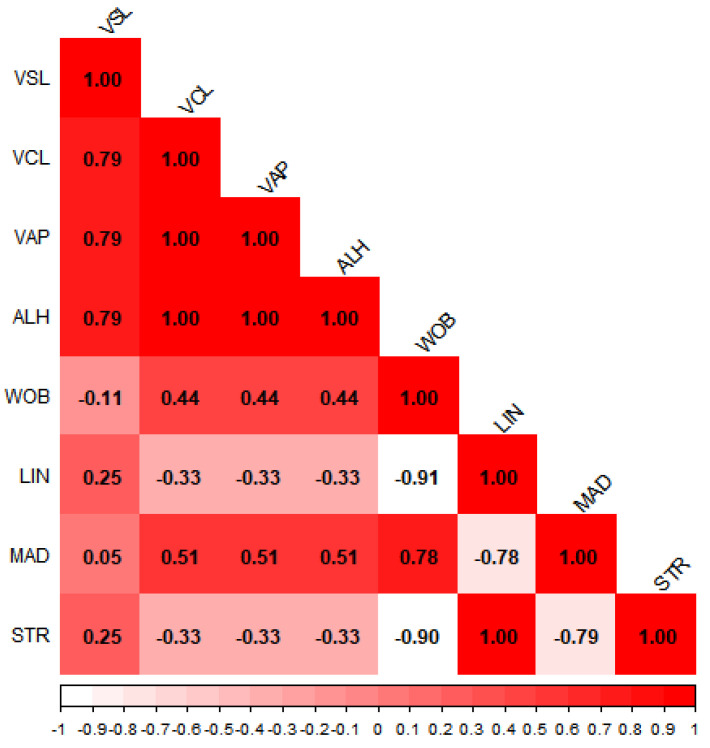
Correlation analysis of sperm motility parameters of Jinding drakes.

Pairwise correlations among the sperm motility traits are summarized in Figure 2. Strong positive correlations (r ≈ 0.79) were observed among the velocity-related parameters (VSL, VCL, VAP, and ALH). WOB was moderately correlated with VCL, VAP, and ALH (r ≈ 0.44), but weakly and negatively correlated with VSL (r ≈ −0.11). LIN and STR were nearly perfectly correlated (r = 1.00) and both were negatively associated with ALH (r ≈ −0.33), suggesting that greater lateral head displacement was associated with reduced path linearity. Similarly, MAD was moderately positively correlated with VCL, VAP, and ALH (r ≈ 0.51) but strongly negatively correlated with LIN and STR (r ≈ −0.78 and −0.79, respectively).

### 3.4. Genomic Regions and Candidate Genes Associated with Sperm Quality Traits

**Figure 3:**
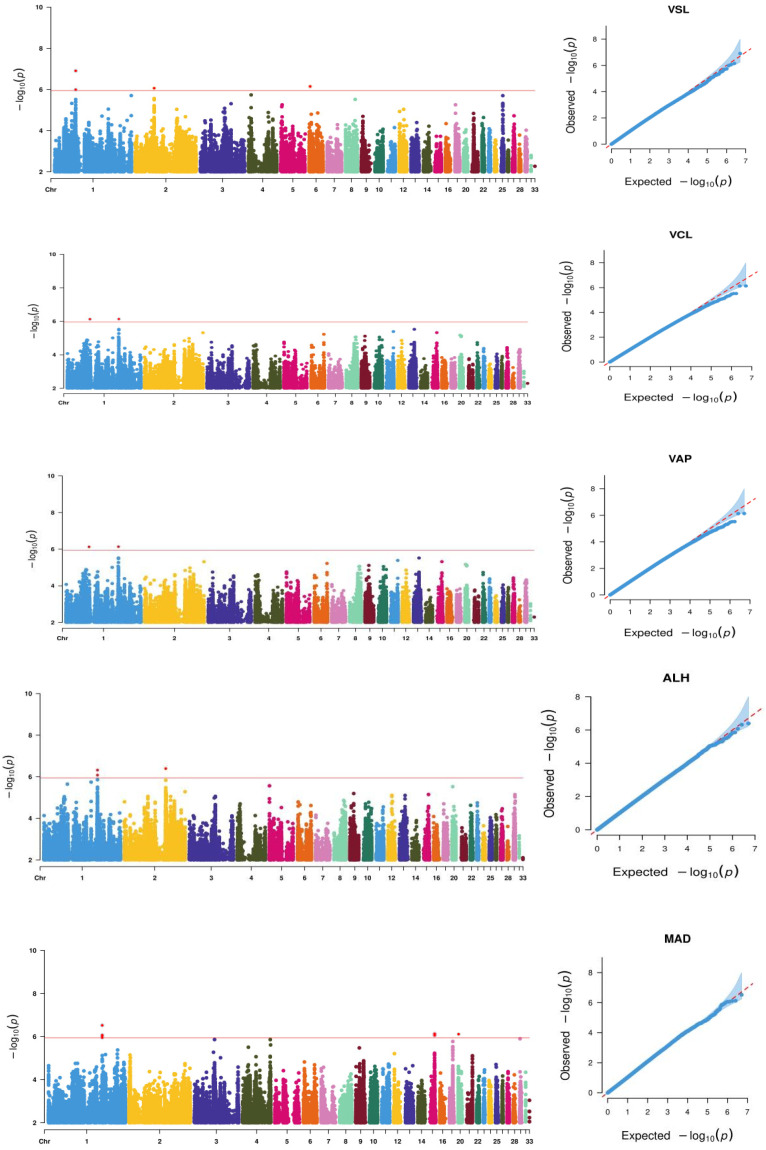
Manhattan plots (**left**) and Q-Q plots (**right**) of genome-wide association studies for sperm motility traits, and significant SNPs passed the genome-wide evidence threshold (red) in Manhattan plots.

After quality control, 886 505 SNPs and 160 samples remained for the GWAS. Then, GWAS identified 15 significant SNPs associated with five sperm motility traits (VSL, VCL, VAP, ALH, and MAD) distributed across chromosomes 1, 2, 6, 15, and 20 (Table 2). No significant associations were detected for WOB, LIN, or STR. The results are visualized in Manhattan plots and Q-Q plots (Figure 3). The significant loci are designated SNP01 to SNP15 based on genomic position.

Among the significant loci, nine were mapped to chromosome 1 and associated with all five motility traits. Notably, both VCL and VAP shared a common locus (ZJU_duck2.0_Chr1_63746049), characterized by a C/T mutation. Similarly, VCL, VAP, and ALH were jointly associated with another locus (ZJU_duck2.0_Chr1_145002302), defined by a G/A mutation. Both regions implicated *Myo16* as a candidate gene influencing sperm motility. For VSL, significant loci were identified on chromosomes 1, 2, and 6, encompassing the candidate genes *Nuak1*, *Ptpn2*, *Psmg2*, *Spire1*, and *Pla2g12b*. The MAD trait was associated with multiple loci on chromosomes 1, 15, and 20, involving the genes *Myo16*, *Usp22*, *Tnfrsf13b*, *Flcn*, and *Msi2*. Collectively, these findings underscore chromosome 1 as a genomic hotspot for sperm motility regulation and highlight *Myo16* as a prominent candidate gene potentially governing sperm movement and structural dynamics.

### 3.5. Genotypic Distribution of Significant SNPs

The genotypic distributions of the 15 SNPs significantly associated with sperm motility traits are summarized in Table 3. All loci were polymorphic, with genotype frequencies varying considerably across the population. Several loci, including SNP03, SNP8, SNP13, and SNP14, exhibited a skewed distribution where one homozygous genotype was predominant. The complete genotype counts for all significant SNPs are provided in Table 3.

### 3.6. Functional Enrichment Analysis of Candidate Genes Associated with Sperm Quality Traits

**Figure 4:**
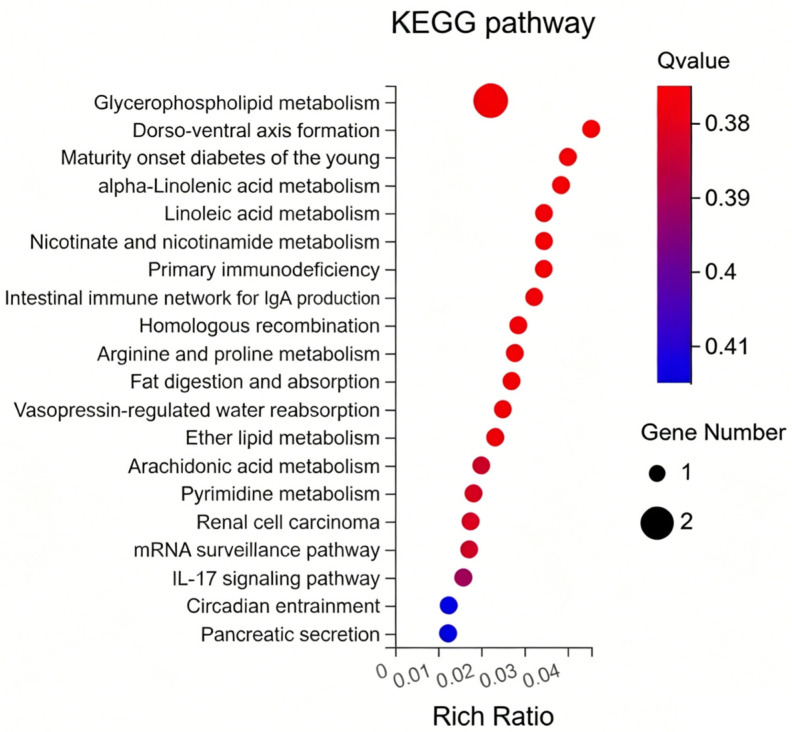
KEGG functional enrichment analysis of the candidate genes associated with sperm quality traits.

Pathway enrichment analysis of the candidate genes in reference to the KEGG revealed significant overrepresentation of metabolic pathways, although pathways explicitly labeled as “sperm motility” were not directly enriched. The significantly enriched terms (Figure 4) were predominantly associated with lipid metabolism (e.g., glycerophospholipid, linoleic acid, and arachidonic acid metabolism), in addition to amino acid and nucleotide metabolism (e.g., arginine, proline, and pyrimidine metabolism).

## 4. Discussion

Semen evaluation is especially important to assess male fertility and reproductive potential [6]. Sperm motility is a measure of sperm functionality and movement capacity. Mass sperm motility has been correlated to the reproductive efficiency of sheep [7], poultry [8], and other species. Sperm motility parameters are key indicators of sperm motility and fertilization potential. At present, the CASA system is the most commonly used method to accurately assess sperm motility parameters. A prior study confirmed the accuracy of the CASA system to efficiently assess the motility parameters of ostrich sperm [9]. In the present study, the sperm motility parameters of Jinding drakes, as determined with the CASA system, exhibited moderate variation, with CVs ranging roughly from 4% for WOB to 9% for MAD (Table 1). Such variability is typical for livestock semen traits and suggests substantial genetic contribution [4,10]. For example, similar moderate heritabilities were reported for boar sperm motility and counts [10], while a GWAS of dairy cattle indicated low to moderate heritability (≈0.04–0.30) for motility [4].

PCA of the eight motility traits analyzed in the present study revealed wide dispersion without clear clustering by subgroup, indicating no difference in semen motility parameters among the studied individual drakes. The lack of distinct clusters suggests a complex, polygenic architecture rather than population structure effects [10,11]. Overall, the PCA results indicate that the differences in major sperm motility traits among samples are mainly driven by a few key indicators. However, no significant natural clustering patterns were observed, suggesting that these indicators are continuously distributed in the population, providing a basis for subsequent analyses of genetic variation and association. Although PCA showed no obvious population clustering among experimental individuals, subtle genetic stratification could not be entirely ruled out.

In a previous study of ostrich semen, samples with higher motility scores also exhibited higher VCL and VAP, which are associated with fertility [9]. In this study, velocity parameters VSL, VCL and VAP were strongly positively correlated (r ≈ 0.8). WOB was moderately positively correlated with VCL, VAP and ALH, but weakly negatively correlated with VSL, reflecting its role in characterizing path irregularity. LIN and STR were nearly identical (r ≈ 1.00) and both negatively correlated with ALH and MAD. It is consistent with the idea that larger lateral head displacement (ALH/MAD) is associated with reduced linearity. These patterns are qualitatively similar to reports of other species. For example, the motility parameters of post-thaw boar sperm are also reportedly strongly inter-correlated [11], while a GWAS noted overlapping quantitative trait loci for average path velocity and total motility of bovine sperm [12]. Thus, our correlation results align with established CASA trait relationships that velocity measures co-vary due to shared physiology, and increased head oscillation tends to reduce straight-line movement, as reported across species [11].

Our GWAS identified 15 SNPs significantly associated with five of the eight traits (VSL, VCL, VAP, ALH, MAD) at a genome-wide *p*-value threshold of 10^−6^ (Figure 3), as reported for other livestock [10]. Strikingly, chromosome 1 coded for nine of the 15 significant SNPs, with multiple traits mapped to overlapping positions. Several markers (e.g., ZJU_duck2.0_Chr1_63746049 and ZJU_duck2.0_Chr1_145002302) influenced both VCL and VAP (and ALH), reflecting pleiotropic effects. Overlapping associations across velocity traits are common in semen GWAS. For instance, pig studies identified the same quantitative trait loci affecting motility and velocity traits (e.g., *STRA8* and *CATSPER1* with motility and progressive motility) [13]. Similarly, a GWAS of boar (Pietrain) found 36 window regions related to one or multiple semen traits across 19 chromosomes [4]. Thus, the shared SNPs on chromosome 1 suggest a core set of genes that control the speed and pattern of sperm movement.

Our GWAS revealed several promising candidate genes linked to the sperm motility traits of ducks. Foremost among these is *Myo16*, located within the major effect region of chromosome 1. This gene, which encodes an unconventional myosin motor protein, is associated with VCL, VAP, and ALH. Although not previously implicated in fertility, *Myo16* is potentially linked to regulation of the actin cytoskeleton during spermiogenesis, analogous to *Myo6* [4,10], thereby positioning *Myo16* as a novel and potential candidate gene for functional validation in avian reproduction.

VSL is a key indicator of the capacity of sperm to move in a straight line. Higher VSL values indicate stronger straight-line motility, which is crucial for sperm to move forward in the female reproductive tract. Our GWAS identified four specific SNPs associated with VSL on chromosomes 1, 2, and 6, which were annotated to 19 candidate genes, including *Nuak1*. Notably, the C/T mutation at position 51111549 on chromosome 2 exhibited significant genotype–phenotype differences in the study population, with dominance of the CC genotype. The candidate genes at this locus include *Ptpn2*, *Psmg2*, *Cep76*, and *Spire1*. Notably, *Ptpn2* has been linked to cryopreservation tolerance [2], while *Cep76* is critical for sperm flagellar assembly and motility, with mutations leading to the loss of key structural proteins [3,14]. *Spire1*, an actin nucleator, works cooperatively with Formin-2 in the fertilization cone, underscoring the fundamental role of cytoskeletal dynamics in sperm progression [15].

The parameters VCL, VAP, and ALH were closely linked to mutations of several genes and loci, particularly at positions 63746049 (C/T) and 145002302 (G/A) on chromosome 1, and position 112052675 (A/G) on chromosome 2. Notably, the A/G mutation at position 112052675 on chromosome 2 showed a significant genotype distribution difference within the population, with significantly greater dominance of the AA genotype. Candidate genes associated with these loci included *Loc119715977*, *Loc110353471*, and *Loc106018375*, with *Jarid2* and *Trnay-Gua* being key candidate genes involved in sperm motility and regulation. *Jarid2* (Jumonji AT-rich interactive domain 2), a member of the histone demethylase family, plays a crucial role in spermatogenesis by regulating methylation of histone H3K27 through interaction with the PRC2 complex. This modification is essential for gene silencing and epigenetic regulation during embryonic development and germ cell differentiation, thereby influencing sperm motility and fertility [16,17].

Overall, seven SNPs on chromosomes 1, 15, and 20 were associated with the MAD parameter. Candidate genes at these loci include *Usp22*, *Tnfrsf13b*, *Pld6*, *Flcn*, *Cops3*, *Nt5m*, *Med9*, *Rasd1*, and *Pemt*, which are involved in sperm motility and fertility. USP22 expression is upregulated in fertile men and downregulated in those with oligospermia, highlighting its importance in maintaining normal sperm production [18]. *Tnfrsf13b* (TACI), a receptor involved in immune regulation, impacts fertility through B cell survival and function, with defects potentially leading to infertility [19]. *Pld6* is crucial for the differentiation and maintenance of germline stem cells and gonadal development, with mutations linked to infertility and compromised mitochondrial function in sperm [20,21,22]. *Flcn*, associated with Birt-Hogg-Dubé syndrome, affects testicular development and fertility, with mutations contributing to infertility [23]. *Cops3*, part of the COP9 signalosome complex, plays an essential role in spermatogenesis, whereas overexpression in abnormal testes suggests influence on developmental defects and sperm production [24]. *Rasd1*, regulated by steroid hormones, affects sperm quality by influencing cell proliferation and apoptosis, with reduced expression linked to impaired sperm function in animals exposed to endocrine disruptors [25]. Lastly, *Pemt*, involved in phosphatidylcholine synthesis, is vital for maintenance of sperm membrane integrity and fluidity, with polymorphisms linked to sperm concentration and vitality, emphasizing a crucial role in fertility [26,27].

Further, KEGG functional enrichment analysis of the candidate genes may not act through a canonical direct sperm motility pathway. Instead, it may exert an indirect regulatory effect by fundamental cellular processes, particularly those governing energy metabolism, membrane integrity, and intracellular signaling. These underlying biological processes are essential for sustaining normal sperm physiological function and motility performance.

## 5. Conclusions

This study analyzed the phenotypic variation and genetic architecture of sperm motility traits in Jinding drakes using computer-assisted sperm analysis. The results revealed moderate phenotypic variability across motility parameters, indicative of a polygenic inheritance pattern. Genome-wide association analysis identified 15 significant SNPs associated with five key motility traits (VSL, VCL, VAP, ALH, and MAD), with a notable concentration of loci on chromosome 1, suggesting the presence of a genomic hotspot for sperm motility regulation. Several candidate genes were implicated in these associations, including *Myo16* (cytoskeletal dynamics), *Cep76* (flagellar structure and function), and *Jarid2* (epigenetic regulation during spermatogenesis), as well as *Usp22*, *Pld6*, *Flcn*, *Rasd1*, and *Ptpn2*, which are involved in membrane integrity, mitochondrial activity, and immune regulation. Functional enrichment analysis further indicated that genetic influences on sperm motility are likely mediated through fundamental cellular processes such as lipid metabolism and energy production. However, the relatively small sample size may limit the statistical power of the GWAS; therefore, further studies with larger populations are warranted to validate the identified loci. These findings provide novel insights into the complex genetic basis of sperm motility in ducks and establish a foundation for molecular breeding strategies aimed at improving reproductive efficiency in waterfowl.

## Figures and Tables

**Table 1:** Statistical analysis of the sperm motility parameters of Jinding drakes.

Parameter	VSL, μm/s	VCL, μm/s	VAP, μm/s	ALH, μm	WOB (%)	LIN (%)	MAD (°)	STR (%)
Avg ± SD	24.036 ± 1.975	54.387 ± 3.643	38.286 ± 2.604	15.755 ± 1.055	0.921 ± 0.040	0.454 ± 0.033	655.503 ± 61.276	0.637 ± 0.051
Max	29.389	68.201	46.215	18.463	1.000	0.536	794.963	0.760
Min	18.638	42.145	32.241	13.172	0.811	0.371	470.941	0.512
CV (%)	8.215	6.698	6.800	6.699	4.299	7.176	9.348	7.937

Abbreviations: VSL, straight-line velocity; VCL, curvilinear velocity; VAP, average path velocity; ALH, amplitude of lateral head displacement; MAD, angular displacement; WOB, wobble; LIN, linearity; and STR, straightness.

**Table 2:** Significant SNPs and candidate genes related to sperm motility traits of Jinding drakes.

Trait	SNP	*p*	*β* Values	Genotype	Candidate Genes	SNP No.
VSL	ZJU_duck2.0_Chr1_56978308	1.02 × 10^−6^	0.32 ± 0.08	G/C	NUAK1, C1h12orf75	SNP01
	ZJU_duck2.0_Chr1_56978387	1.24 × 10^−7^	−0.29 ± 0.07	T/C		SNP02
	ZJU_duck2.0_Chr2_51111549	8.63 × 10^−7^	0.41 ± 0.09	C/T	Loc119716042, Ptpn2, Psmg2, Cep76, Spire1	SNP03
	ZJU_duck2.0_Chr6_2934713	7.12 × 10^−7^	−0.35 ± 0.08	G/A	Fam149b1, Ecd, Nudt13, Loc119717346, P4ha1, Loc119717344, Loc119717345, Pla2g12b, Loc113844055, Oit3, Mcu, Micu1	SNP04
VCL	ZJU_duck2.0_Chr1_63746049	7.48 × 10^−7^	0.27 ± 0.07	C/T	Tubgcp3, Myo16	SNP05
	ZJU_duck2.0_Chr1_145002302	7.36 × 10^−7^	−0.38 ± 0.09	G/A	Wnk1, Loc119714359, Rad52, Loc106018272, Erc1	SNP06
VAP	ZJU_duck2.0_Chr1_63746049	7.48 × 10^−7^	0.28 ± 0.07	C/T	Tubgcp3, Myo16	SNP05
	ZJU_duck2.0_Chr1_145002302	7.33 × 10^−7^	−0.37 ± 0.08	G/A	Wnk1, Loc119714359, Rad52, Loc106018272, Erc1	SNP06
ALH	ZJU_duck2.0_Chr1_145002302	4.78 × 10^−7^	0.39 ± 0.09	G/A	Tubgcp3, Myo16	SNP06
	ZJU_duck2.0_Chr1_145005645	8.35 × 10^−7^	−0.26 ± 0.06	C/G		SNP07
	ZJU_duck2.0_Chr2_112052675	4.05 × 10^−7^	0.43 ± 0.10	A/G	Jarid2, Loc119715977, Trnay-Gua, Loc110353471, Loc106018375	SNP08
MAD	ZJU_duck2.0_Chr1_145148615	2.99 × 10^−7^	−0.45 ± 0.10	G/A	Myo16	SNP09
	ZJU_duck2.0_Chr1_145148754	9.41 × 10^−7^	0.30 ± 0.07	G/T		SNP10
	ZJU_duck2.0_Chr1_145148918	8.53 × 10^−7^	−0.25 ± 0.06	C/T		SNP11
	ZJU_duck2.0_Chr1_145148965	1.11 × 10^−6^	0.24 ± 0.06	A/T		SNP12
	ZJU_duck2.0_Chr15_14634290	8.67 × 10^−7^	−0.33 ± 0.08	G/T	Usp22, Tnfrsf13b, Loc101792826, Loc119718466, Pld6, Flcn, Cops3, Nt5m, Med9, Rasd1, Pemt, Loc119718417	SNP13
	ZJU_duck2.0_Chr15_14635438	7.44 × 10^−7^	0.36 ± 0.08	T/A		SNP14
	ZJU_duck2.0_Chr20_1543956	7.70 × 10^−7^	−0.31 ± 0.07	C/T	Msi2, Mrps23, Cuedc1, Vezf1, Loc119713253, Srsf1, Dynll2, Heatr6, Hnf1b	SNP15

**Table 3:** The sample size of different genotypes at 15 SNPs.

SNP	Genotype and Counts			SNP	Genotype and Counts			SNP	Genotype and Counts		
SNP01	GG	GC	CC	SNP02	TT	TC	CC	SNP03	CC	TT	CT
	29	51	18		29	51	18		102	3	17
SNP04	GG	AA	GA	SNP05	CC	TT	TC	SNP06	GG	AA	GA
	65	20	43		42	15	41		81	10	27
SNP07	CC	GG	CG	SNP08	AA	GG	AG	SNP09	GG	AA	GA
	60	25	43		107	1	15		90	13	35
SNP10	GG	TT	GT	SNP11	CC	TT	CT	SNP12	AA	TT	AT
	90	14	34		91	15	32		92	15	31
SNP13	GG	TT	GT	SNP14	TT	AA	TA	SNP15	CC	TT	CT
	105	6	27		106	6	27		55	35	48

Note: Values represent the counts of each genotype for individuals passing genotype quality control. Minor missing genotypes at specific markers were excluded, so the total number of valid individuals per SNP may be slightly less than the full sample size of 160.

## Data Availability

All data generated or analyzed during this study are included in this published paper.
